# An Efficient Model for Leafy Vegetable Disease Detection and Segmentation Based on Few-Shot Learning Framework and Prototype Attention Mechanism

**DOI:** 10.3390/plants14050760

**Published:** 2025-03-01

**Authors:** Tong Hai, Yuxin Shao, Xiyan Zhang, Guangqi Yuan, Ruihao Jia, Zhengjie Fu, Xiaohan Wu, Xinjin Ge, Yihong Song, Min Dong, Shuo Yan

**Affiliations:** 1 China Agricultural University, Beijing 100083, China; 2022308130304@cau.edu.cn (T.H.); shaoyuxin22@cau.edu.cn (Y.S.); xiyanzhang@cau.edu.cn (X.Z.); ygq2121@cau.edu.cn (G.Y.); jrh24@cau.edu.cn (R.J.); zhengjiefu24@cau.edu.cn (Z.F.); wuxiaohan24@cau.edu.cn (X.W.); gexinjin22@cau.edu.cn (X.G.); songyih2019@163.com (Y.S.); 2 School of English and International Studies, Beijing Foreign Studies University, Beijing 100193, China

**Keywords:** artificial intelligence in agriculture, smart agriculture, disease detection, few-shot learning

## Abstract

This study proposes a model for leafy vegetable disease detection and segmentation based on a few-shot learning framework and a prototype attention mechanism, with the aim of addressing the challenges of complex backgrounds and few-shot problems. Experimental results show that the proposed method performs excellently in both object detection and semantic segmentation tasks. In the object detection task, the model achieves a precision of 0.93, recall of 0.90, accuracy of 0.91, mAP@50 of 0.91, and mAP@75 of 0.90. In the semantic segmentation task, the precision is 0.95, recall is 0.92, accuracy is 0.93, mAP@50 is 0.92, and mAP@75 is 0.92. These results show that the proposed method significantly outperforms the traditional methods, such as YOLOv10 and TinySegformer, validating the advantages of the prototype attention mechanism in enhancing model robustness and fine-grained feature expression. Furthermore, the prototype loss function, which optimizes the distance relationship between samples and category prototypes, significantly improves the model’s ability to discriminate between categories. The proposed method shows great potential in agricultural disease detection, particularly in scenarios with few samples and complex backgrounds, offering broad application prospects.

## 1. Introduction

With the continuous growth of the global population and the impact of climate change, agricultural production is facing increasingly severe challenges [1,2,3]. In the northern regions of China, climate change has exacerbated the frequency of crop diseases, posing significant threats to agricultural productivity [4]. Leafy vegetables, as one of the essential categories of vegetables in China, are particularly susceptible to diseases due to their complex growing environments and diverse types of pathogens, which significantly affect both yield and quality [5,6,7]. The accurate and timely detection and prevention of diseases are crucial for ensuring agricultural productivity, enhancing crop yields, and maintaining food safety [8]. Traditionally, disease detection has relied on manual inspection and diagnosis [9]. However, such methods are labor-intensive, require significant technical expertise, and are influenced by subjective factors, making it challenging to detect potential diseases promptly, thereby missing the optimal time for intervention [10].

With the modernization of agricultural practices, the limitations of traditional disease detection methods have become increasingly apparent, especially in large-scale agricultural production systems where manual inspection is no longer sufficient to meet the demands [11]. Therefore, an automated and accurate disease detection approach is urgently needed to improve efficiency and ensure precision. In recent years, deep learning technologies have achieved remarkable breakthroughs in computer vision and demonstrated significant potential in agricultural disease detection [12]. By training deep neural networks, computers can automatically extract features from images and identify and classify different types of diseases, offering unparalleled advantages over traditional methods [13]. Consequently, the application of deep learning-based methods for disease detection and segmentation in agriculture has become a prominent research focus in both academic and industrial fields [14].

With the advancements in computer vision technologies, image-processing-based disease detection methods have emerged [15]. These methods primarily rely on extracting image features such as color, texture, and shape to identify diseased areas [16]. Common image processing techniques include threshold segmentation, edge detection, and texture analysis [17]. Although these methods can achieve satisfactory results in simple scenarios, they depend heavily on manually defined features and rules, making them inadequate for complex and dynamic agricultural environments. Particularly in situations where diseases are diverse and environmental conditions fluctuate significantly, traditional image processing methods often fail to effectively differentiate between diseased and healthy plants, resulting in low detection accuracy [18]. Additionally, traditional methods exhibit poor adaptability in scenarios involving multiple diseases or diverse planting conditions. In cases of limited data or imbalanced disease types, the robustness and generalization capabilities of these models are weak [19]. These limitations present substantial challenges for practical applications, necessitating the development of novel technological solutions.

The early detection and rapid control of leafy vegetable diseases are critical for enhancing agricultural productivity [20]. Timely and accurate disease detection can not only prevent the spread of diseases but also reduce pesticide usage, minimize environmental pollution, and ensure the healthy growth of crops, thereby improving the quality and yield of agricultural products [21]. Early detection provides farmers with scientific decision-making support, preventing disease outbreaks and reducing economic losses. Currently, disease control for leafy vegetables primarily relies on manual inspection and chemical treatment [22]. While technologies such as remote sensing, drones, and ground-based sensors are being used to assist in monitoring, these methods remain in the preliminary stages of application and face challenges such as incomplete data collection, low accuracy, and operational complexity [23]. In many rural areas, agricultural infrastructure is underdeveloped, and the application of information technology is not widespread. Therefore, efficient and intelligent methods for detecting leafy vegetable diseases hold significant application value.

In recent years, the successful application of deep learning techniques, particularly convolutional neural networks (CNNs), in computer vision has provided new solutions for intelligent agricultural scenarios [24,25]. Compared to traditional image processing methods, deep learning can automatically learn effective feature representations from large datasets, reducing the need for manual feature design. Specifically, deep learning models have demonstrated exceptional performance in tasks such as image classification, object detection, and semantic segmentation. Abdu et al. proposed a plant disease identification method that optimally extracts global and local feature descriptors representing lesions, reducing feature redundancy and vector length to improve performance and thus achieving high precision and a recall rate exceeding 99% [26]. Rahman et al. introduced an image-processing-based technique for the automatic detection and treatment of tomato leaf diseases, calculating 13 statistical features from tomato leaves and using a support vector machine (SVM) to classify diseases, achieving an accuracy exceeding 85% [27]. Li et al. developed an improved vegetable disease detection algorithm based on YOLOv5s, modifying the CSP, FPN, and NMS modules within YOLOv5s to mitigate the impact of environmental factors, enhance multi-scale feature extraction capabilities, and improve detection performance, achieving a mean average precision (mAP) of 93.1% for vegetable disease detection [28]. Tiwari et al. proposed a potato disease detection model utilizing transfer learning to extract relevant features from datasets, achieving an accuracy of 97.8% [29]. Wang et al. presented a two-stage model for cucumber leaf disease severity classification under complex backgrounds, combining DeepLabV3+ and U-Net. The leaf segmentation accuracy reached 93.27%, the lesion segmentation’s Dice coefficient was 0.6914, and the average accuracy for disease severity classification was 92.85% [30]. Jiang et al. used a CNN to extract features from rice leaf disease images and applied an SVM for the classification and prediction of specific diseases, achieving an average accuracy of 96.8% [31].

While these deep learning-based models demonstrate strong performance, they typically require large-scale labeled datasets and are not designed to handle new disease classes with minimal data. Few-shot learning approaches in agriculture have attempted to address this issue by introducing meta-learning frameworks that learn from limited samples. However, the existing FSL-based methods often struggle with distinguishing visually similar diseases or segmenting diseased regions precisely due to feature extraction limitations. To address the limitations of the traditional disease detection methods and the challenges of deep learning in practical applications, this study proposes a disease detection and segmentation model based on few-shot learning. The proposed method is particularly suited for monitoring diseases in leafy vegetables, enabling accurate detection and segmentation with limited disease samples. The primary innovations of this study are as follows:

- Few-shot learning framework: To address the scarcity of agricultural disease data samples, a few-shot learning (FSL) network architecture is introduced. By incorporating a prototype extraction module and prototype attention mechanism, the rapid and accurate identification and segmentation of diseased areas are achieved under limited data conditions.
- Dual-task network design: A dual-task network structure is designed to combine object detection and semantic segmentation tasks, simultaneously performing disease localization and fine-grained segmentation, thereby enhancing detection accuracy and segmentation performance.
- Integration of prototype extraction and attention mechanism: A prototype-based attention mechanism is proposed to guide the model in focusing on the key features of diseased areas, effectively improving learning capabilities under low-sample conditions.
- Practical applicability: Considering the practical application requirements, the model’s precision, computational efficiency, and deployment are optimized. The proposed method enables real-time disease detection and control in vegetable-growing regions, aiding farmers in efficient disease management.

Through these innovations, the proposed disease detection and segmentation model is applicable to various regions, enabling the efficient and precise automated detection and control of leafy vegetable diseases, with significant theoretical and practical implications.

## 2. Related Work

### 2.1. Few-Shot Learning

FSL refers to the ability to extract useful features from a limited number of samples, enabling effective classification or recognition [32,33]. The traditional deep learning methods typically rely on large amounts of annotated data for training. However, in real-world applications, especially in agriculture, the cost and time required for obtaining extensive labeled datasets are substantial [34]. FSL leverages techniques such as transfer learning and meta-learning to achieve strong performance even in data-scarce scenarios [35,36]. The key to FSL lies in utilizing limited samples to facilitate effective knowledge transfer, thereby enhancing the model’s generalization capability.

One of the most common strategies in FSL is prototype-based learning. This approach classifies each sample by mapping it to a “prototype” vector for each class. Under this framework, given an input sample *x*, the corresponding class prototype *C* is determined by calculating the distance between the input sample and each class prototype. In Euclidean space, the prototype $C_{i}$ is typically obtained as the mean of all samples within the class, as described by the following equation:

(1) $$C_{i}=\frac{1}{N_{i}}\sum_{x_{j}\in C_{i}}x_{j}$$

where $C_{i}$ represents the prototype for class *i*, $N_{i}$ denotes the number of samples in class *i*, and $x_{j}$ is the *j*-th sample in class *i*. For a new input sample *x*, its class is determined based on the distance to each prototype, typically measured using the Euclidean distance, as given by the following:

(2) $$d\left(x,C_{i}\right)=\left|x-C_{i}\right|$$

where $d(x,C_{i})$ is the distance between sample *x* and prototype $C_{i}$. The objective of the model in FSL is to establish a classifier with strong generalization capabilities using a limited number of training samples. In agricultural disease detection, FSL effectively utilizes limited disease samples to improve the model’s ability to identify different types of diseases [37]. In agricultural environments, particularly for rare or newly encountered diseases, acquiring a large number of samples for training is often infeasible, making FSL especially valuable. By employing FSL, disease detection models can achieve high accuracy and robust generalization even with limited training data, which is crucial for large-scale agricultural monitoring and disease prevention [36,38].

### 2.2. Object Detection Tasks

Object detection is a critical task in the field of computer vision, aimed at identifying all the objects of interest in a given image or video and precisely locating them using bounding boxes. Object detection requires not only the recognition of the object categories but also the accurate marking of their positions, making it a multi-task learning problem that involves both classification and regression. In recent years, significant breakthroughs have been achieved in object detection with the application of deep learning methods, particularly CNNs, enabling the widespread adoption of this technology in various domains, including agriculture, transportation, and security.

The fundamental principle of object detection involves scanning each region of an image using algorithms to determine whether it contains a target object. If a target object is present, the system outputs the object’s category and its location, typically represented by a bounding box [39]. Specifically, the output of object detection includes the following two pieces of information: the object’s category label (e.g., “apple”, “carrot”, etc.) and its location, usually represented by a rectangle defined by four parameters $\left(x_{min},y_{min},x_{max},y_{max}\right)$, corresponding to the top-left and bottom-right coordinates of the bounding box. Object detection tasks can be divided into two components—object classification and location regression. Object classification determines whether a specific target object exists within each region of the image, while location regression identifies the precise position of the object [40]. Under the deep learning framework, object detection models are primarily based on CNNs, incorporating techniques such as region extraction, candidate box generation, and non-maximum suppression (NMS) to achieve accurate object detection.

The mathematical formulations for object detection typically involve two key steps—bounding box regression and category classification. For bounding box regression, the object detection model extracts features from the image through convolutional layers and predicts the object’s location using regression calculations. The loss function for bounding box regression is often based on the $L_{2}$ norm and is expressed as follows:

(3) $$L_{\mathrm{bbox}}=\sum_{i=1}^{N}{\left({\hat{x}}_{i}-x_{i}\right)}^{2}+{\left({\hat{y}}_{i}-y_{i}\right)}^{2}+{\left({\hat{w}}_{i}-w_{i}\right)}^{2}+{\left({\hat{h}}_{i}-h_{i}\right)}^{2}$$

where $x_{i}$, $y_{i}$, $w_{i}$, and $h_{i}$ represent the ground truth coordinates and dimensions of the bounding box, while ${\hat{x}}_{i}$, ${\hat{y}}_{i}$, ${\hat{w}}_{i}$, and ${\hat{h}}_{i}$ denote the model-predicted coordinates and dimensions. This loss function measures the discrepancy between predicted and ground truth boxes, enabling the model to learn accurate object localization. For object classification, the model assigns a category label to each candidate box. The classification loss is typically cross-entropy loss, which quantifies the difference between the predicted category probabilities and the ground truth labels. The formula for cross-entropy loss is as follows:

(4) $$L_{\mathrm{cls}}=-\sum_{i=1}^{N}\left(y_{i}\cdot \log\left({\hat{y}}_{i}\right)+\left(1-y_{i}\right)\cdot \log\left(1-{\hat{y}}_{i}\right)\right)$$

where $y_{i}$ is the ground truth label (usually 0 or 1), and ${\hat{y}}_{i}$ is the model-predicted probability. This loss function helps the model minimize the discrepancy between predictions and true labels, enabling accurate object classification. The application of object detection in agriculture has expanded significantly, particularly in crop disease detection, crop growth monitoring, and agricultural robotics. Agricultural environments present challenges such as complex backgrounds, occlusions, and variations in lighting. Object detection methods have been widely used to automate the detection and identification of crop diseases. For instance, high-resolution cameras installed in fields, combined with object detection technology, can automatically identify diseased areas and annotate their locations, enabling timely preventive measures. In early-stage disease detection, object detection methods help farmers identify potential risks in advance, reducing crop losses.

A notable application involves using YOLO (You Only Look Once) models for crop disease detection. In crop management, object detection is utilized for monitoring growth conditions and enabling precision management [41]. These tasks often involve processing large volumes of image data in complex field environments, requiring robust models capable of handling interference [42]. The potential of object detection in agriculture is vast, offering opportunities to improve automation in agricultural production and support tasks such as disease monitoring, crop management, and agricultural robotics. However, the complexity and variability of agricultural environments pose challenges, including complex backgrounds, object diversity, occlusions, and lighting variations. Addressing these challenges and improving the robustness and adaptability of object detection models remain pressing research issues.

### 2.3. Semantic Segmentation

Semantic segmentation is a type of image segmentation task that assigns each pixel in an image to a predefined category [43]. In agricultural disease detection, semantic segmentation is used to isolate diseased areas from the background, enabling the precise annotation of the affected regions [44]. The objective of semantic segmentation is to label every pixel in the image with its corresponding class. Traditional methods, such as threshold-based segmentation and region-growing algorithms, often struggle with noise and complex backgrounds, requiring manual feature design and lacking the ability to learn effective feature representations automatically.

Deep learning-based semantic segmentation typically employs CNNs as the foundational architecture. Representative methods include fully convolutional networks (FCNs), SegNet, and U-Net [45,46,47]. These methods effectively extract contextual information from images, enabling accurate segmentation. U-Net is a classic semantic segmentation network characterized by a symmetric encoder–decoder structure that preserves spatial information during the segmentation process. The basic U-Net architecture is described as follows:

(5) y=Decoder(Encoder(x))

where *x* is the input image, Encoder represents the encoder responsible for feature extraction, Decoder represents the decoder that restores the features to the original image size, and *y* is the segmentation output. In agriculture, semantic segmentation has been widely applied to tasks such as crop health monitoring, disease detection, and weed identification [48]. In leafy vegetable disease detection, semantic segmentation accurately separates the diseased areas from the healthy regions, enabling precise localization and analysis. For instance, the Mask R-CNN model, a significant advancement in semantic segmentation, integrates object detection and pixel-level segmentation, making it particularly valuable for accurately segmenting diseases in leafy vegetables [49].

In summary, object detection and semantic segmentation play pivotal roles in agricultural disease detection. By integrating deep learning technologies, especially CNNs and Transformer models, disease detection systems can achieve the precise identification and segmentation of diseases, significantly enhancing the efficiency of agricultural disease management. The application of deep learning technologies substantially automates disease detection processes, helping farmers minimize disease-related losses and ensuring the stability and sustainability of agricultural production.

## 3. Results and Discussion

### 3.1. Disease Detection Results

The objective of this experiment was to compare the performance of mainstream detection and segmentation models and validate the effectiveness of the proposed model in the tasks of leafy vegetable disease detection and segmentation. The evaluation metrics included precision, recall, accuracy, mAP@50, and mAP@75, providing a comprehensive assessment of the models’ accuracy and robustness for the object detection and segmentation tasks. The experimental results are summarized in Table 1 and Table 2, which highlight the performance of different models in disease detection and segmentation. As shown in the tables, models with strong multi-scale feature modeling capabilities, such as YOLOv10 and TinySegformer, outperformed their predecessors, while the proposed method surpassed all the existing models across all metrics, demonstrating significant advantages in complex backgrounds and few-shot scenarios.

Specifically, in the object detection task, YOLOv10 achieved mAP@50 and mAP@75 scores of 0.87 and 0.86, respectively, showing notable improvement over DETR and YOLOv9. This improvement can be attributed to YOLOv10’s optimized detection head and multi-scale feature fusion module, which enhanced its capability to detect small objects. However, the proposed method achieved mAP@50 and mAP@75 scores of 0.91 and 0.90, respectively, significantly outperforming YOLOv10. This highlights the effectiveness of the proposed prototype extraction module and prototype attention mechanism in optimizing few-shot feature representation and focusing on critical regions. In the segmentation task, TinySegformer, leveraging its Transformer architecture, exhibited strong global feature modeling capabilities, with mAP@50 and mAP@75 scores of 0.91 and 0.90, respectively. Nonetheless, the proposed method further improved these metrics to 0.92 and achieved precision and recall scores of 0.95 and 0.92, respectively, demonstrating its comprehensive superiority in accuracy and robustness. From a mathematical perspective, DETR’s self-attention mechanism facilitates global correlations among features, but its high computational complexity and limited small-object detection capability led to relatively weaker performance in recall. In contrast, YOLO models achieved significant reductions in computational overhead through structural optimization and lightweight design, while enhancing robustness via multi-scale feature pyramids. TinySegformer leveraged a lightweight Transformer architecture to improve the capture of contextual information, although its feature extraction was still constrained by the resolution capacity of multi-head attention. The proposed method combined the prototype extraction module and prototype attention mechanism to significantly enhance intra-class compactness and inter-class separability in the feature space. Furthermore, the prototype-based loss function explicitly constrained the distance relationships between the samples and the prototypes during optimization, thereby improving class discrimination and suppressing background noise in both the detection and segmentation tasks. These mathematical characteristics ensured the superior performance of the proposed method in challenging agricultural scenarios with limited samples, enabling it to achieve leading experimental results in both object detection and semantic segmentation tasks.

### 3.2. Discussion of Challenging Cases

The objective of this experiment was to analyze the performance of the proposed model in detecting different types of diseases, especially under complex backgrounds or with confusing features, and to evaluate the model’s ability to handle challenging cases. By comparing the detection results of various diseases such as downy mildew, gray mold, black rot, sclerotinia, and brown spot, the experiment aimed to reveal the strengths and weaknesses of the model in the fine-grained discrimination and few-shot disease categories. The detection results for each disease type are shown in Table 3.

It can be seen that the proposed model performs well across all the disease types, with downy mildew achieving a precision of 0.96 and mAP@50 of 0.94, indicating high accuracy in detecting diseases with clear features. However, for disease types with complex backgrounds or similar features, such as brown spot and sclerotinia, although the model still outperforms existing methods, the mAP@75 slightly decreases, highlighting the challenge these types of diseases pose to the model’s discriminative ability. From the experimental results, the high performance of the model on diseases like downy mildew can be attributed to the distinctive features of these diseases, such as the white powdery spots of downy mildew and the decaying areas of gray mold. These features exhibit strong separability in the feature space. The model captures these features through the prototype extraction module.

### 3.3. Ablation Study on Different Attention Mechanisms

The objective of this experiment was to analyze the impact of different attention mechanisms on model performance, particularly in the tasks of leafy vegetable disease detection and segmentation. The following three different attention mechanisms were evaluated in the experiment: standard self-attention, the convolutional block attention module (CBAM), and the proposed prototype attention mechanism. By comparing the performances of these models in terms of precision, recall, accuracy, mAP@50, and mAP@75, the aim was to reveal how the different attention mechanisms contribute to enhancing feature expression, improving the robustness of the model, and increasing detection accuracy. The experimental results are shown in Table 4, where significant differences in performance across the three methods are observed.

From the experimental results, it can be observed that the standard self-attention mechanism shows relatively low performance across precision, recall, accuracy, mAP@50, and mAP@75, with values of 0.73, 0.70, 0.72, 0.72, and 0.71, respectively. This is primarily because while self-attention can capture global features, it has limitations when processing complex backgrounds and fine-grained distinctions. Self-attention calculates the relationships between all the positions to obtain global information, but it fails to effectively suppress noise and irrelevant backgrounds, leading to weaker discriminative power in detailed and complex scenarios. On the other hand, the CBAM, which introduces both spatial and channel-wise attention mechanisms based on standard self-attention, improves the model’s performance in complex backgrounds by focusing on important spatial regions and feature channels. CBAM demonstrates a significant improvement in all the evaluation metrics, with precision at 0.85, recall at 0.81, accuracy at 0.83, mAP@50 at 0.84, and mAP@75 at 0.83. This indicates CBAM’s advantage in capturing important features, particularly when dealing with various backgrounds and interfering diseases, and it effectively enhances detection accuracy and robustness. However, the proposed prototype attention mechanism outperforms all the other models in every evaluation metric, achieving precision of 0.95, recall of 0.92, accuracy of 0.93, mAP@50 of 0.92, and mAP@75 of 0.92. The prototype attention mechanism combines the advantages of prototype extraction and adaptive attention weighting, significantly enhancing the model’s discriminative power and focusing ability. Through the prototype extraction module, the model effectively generates global prototype vectors for each disease category. These prototype vectors further guide the weighting of the feature map through the attention mechanism thus strengthening the response of disease regions and suppressing background interference. This approach exhibits superior performance in solving the challenges of FSL and complex backgrounds, and it can effectively improve both the precision and robustness of the model. From a mathematical perspective, the prototype attention mechanism calculates the correlation between each position and the class prototype, generating a weight matrix that achieves adaptive focusing on the categories in the feature space. This prototype-based optimization not only enhances class separability but also increases the model’s sensitivity to details. Therefore, this method achieves high detection and segmentation accuracy for all disease types, making it a highly effective approach for the detection and segmentation of leafy vegetable diseases in complex real-world scenarios.

### 3.4. Ablation Study on Different Loss Functions

This experiment aims to compare the impact of different loss functions on model performance, particularly in the tasks of leafy vegetable disease detection and segmentation, through an ablation study. The following three commonly used loss functions are evaluated: Cross-entropy loss, focal loss, and the proposed prototype loss. By comparing the performance of these loss functions in terms of precision, recall, accuracy, mAP@50, and mAP@75, the experiment seeks to reveal how different loss functions affect the model’s training stability, accuracy, and robustness. The experimental results, as shown in Table 5, clearly demonstrate that the prototype loss outperforms both cross-entropy loss and focal loss across all the evaluation metrics.

From the experimental results, it can be observed that the model using cross-entropy loss performs poorly across all the metrics, with precision at 0.67, recall at 0.64, accuracy at 0.65, mAP@50 at 0.66, and mAP@75 at 0.65. This is because while cross-entropy loss is simple and widely applied, it struggles to effectively differentiate between the fine-grained categories, especially in the case of imbalanced or few-shot classes, leading to weak performance in recognizing rare categories. In contrast, the model using focal loss shows significant improvements in precision and recall, with precision at 0.84, recall at 0.80, accuracy at 0.82, mAP@50 at 0.81, and mAP@75 at 0.81. This improvement is due to focal loss placing a greater emphasis on hard-to-classify examples, enhancing the model’s ability to learn difficult categories, especially when handling imbalanced datasets. However, the prototype loss function proposed in this study performs the best, with precision at 0.95, recall at 0.92, accuracy at 0.93, mAP@50 at 0.92, and mAP@75 at 0.92. Prototype loss optimizes the distance relationship between sample embeddings and class prototypes, significantly improving the model’s ability to discriminate few-shot classes while suppressing background interference, thereby enhancing both classification and segmentation accuracy.

### 3.5. Mobile Application Deployment

To address the limited computational resources of mobile devices, a lightweight disease detection and segmentation model is designed and implemented for deployment on mobile platforms. The lightweight deployment process involves model compression, optimization, and efficient inference on mobile devices. By incorporating knowledge distillation techniques, the model’s computational complexity and memory usage are significantly reduced while maintaining superior detection accuracy. The teacher model is the complete FSL-based disease detection and segmentation model, while the student model is derived by compressing the backbone network and scaling down the feature extraction module. The distillation process comprises the following steps: First, the teacher and student models generate the corresponding feature representations from the input images. Let the teacher model’s output features be $T\in R^{H_{t}\times W_{t}\times C_{t}}$ and the student model’s output features be $S\in R^{H_{s}\times W_{s}\times C_{s}}$, where $H_{t},W_{t},C_{t}$ are the feature dimensions of the teacher model, and $H_{s},W_{s},C_{s}$ are the feature dimensions of the student model, typically satisfying $H_{s}<H_{t},W_{s}<W_{t},C_{s}<C_{t}$. To transfer the knowledge from the teacher model to the student model, a feature alignment-based distillation loss function is employed. The teacher features are first mapped to the same dimensions as the student features using a dimensionality reduction mapping function ϕ(·), as follows:

(6) $$T^{\prime }=\phi \left(T\right),\;T^{\prime }\in R^{H_{s}\times W_{s}\times C_{s}}.$$

The mapped teacher features $T^{\prime }$ and the student features S are then used to compute the distillation loss, defined as the Euclidean distance (L2 Loss), as in the following equation:

(7) $$L_{\mathrm{distill}}=\frac{1}{H_{s}W_{s}C_{s}}\sum_{i=1}^{H_{s}}\sum_{j=1}^{W_{s}}\sum_{k=1}^{C_{s}}{\left(T_{i,j,k}^{\prime }-S_{i,j,k}\right)}^{2}.$$

By minimizing $L_{\mathrm{distill}}$, the student model progressively learns feature representations similar to those of the teacher model. Additionally, the student model is optimized with standard task-specific loss functions, including classification loss for object detection $L_{\mathrm{cls}}$, bounding box regression loss $L_{\mathrm{bbox}}$, and mask loss for segmentation $L_{\mathrm{mask}}$. The total optimization objective is expressed as follows:

(8) $$L=\alpha L_{\mathrm{distill}}+\beta L_{\mathrm{cls}}+\gamma L_{\mathrm{bbox}}+\delta L_{\mathrm{mask}},$$

where α,β,γ,δ are weighting hyperparameters that balance the contributions of each loss term. The proposed lightweight model demonstrated significant improvements in the deployment efficiency for the leafy vegetable disease detection tasks. On mobile devices, the inference speed increased by approximately fivefold, while the model size reduced to only 20% of the teacher model’s size. Despite this compression, detection accuracy remained above 92%. This deployment solution provides crucial technological support for intelligent agricultural applications, showcasing its broad potential for practical use.

### 3.6. Future Work

In future work, we plan to validate our proposed model in real-world agricultural environments to further assess its robustness and applicability. Specifically, we will conduct field experiments at the experimental farmland of the Meteorological Bureau in Wuyuan County, Bayannur, Inner Mongolia, where the agricultural conditions are representative of real-world crop cultivation. This region experiences diverse climatic variations, making it an ideal location to evaluate the model’s performance under varying environmental conditions. We will employ UAVs and multispectral cameras to collect real-time images of leafy vegetable diseases and compare the model’s predictions with expert-annotated ground truth data. Additionally, we will refine the few-shot learning strategy to enhance the model’s adaptability to newly emerging diseases with limited labeled data. Moreover, we aim to integrate our approach into an agricultural disease early warning system, incorporating meteorological data and disease detection results to provide farmers with real-time disease monitoring and prevention recommendations. By bridging the gap between research and practical applications, we seek to further improve the model’s reliability and contribute to intelligent agricultural disease management.

## 4. Materials and Methods

### 4.1. Dataset Collection

Dataset collection is a critical step in machine learning research to ensure the effectiveness of model training and the reliability of its final application. In this study, an image database was constructed for leafy vegetable disease detection and segmentation, encompassing various vegetables and their associated diseases. The data were sourced from two main channels—the Science Park of the Western Campus of China Agricultural University in Haidian District, Beijing, and publicly available online resources. First, on-site image acquisition was conducted at the Science Park of China Agricultural University. The leafy vegetables collected included spinach, lettuce, Chinese cabbage, cabbage, and romaine lettuce. These varieties were selected due to their widespread cultivation in China and their susceptibility to multiple diseases. Images were specifically collected for the following diseases: downy mildew, white rust, anthracnose, viral disease, gray mold, soft rot, black rot, black spot, sclerotinia, and powdery mildew. The number of images for each disease ranged from 1000 to 2000, ensuring the diversity and sufficiency of the data. Although healthy leaf samples were not explicitly labeled as a separate category, the dataset inherently contained a substantial number of healthy leaf regions. Since disease symptoms typically appear as localized lesions, large portions of many collected images contained healthy, unaffected leaf areas. These regions were naturally included within the background of the disease images and served as negative samples during model training. Table 6 presents the detailed distribution of images for each disease.

**Figure 1:**
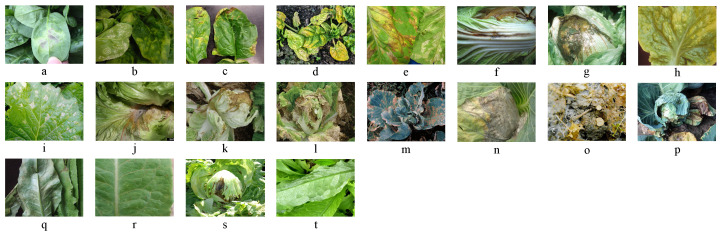
Dateset Samples: (**a**) is spinach downy mildew; (**b**) is spinach white rust; (**c**) is spinach anthrax; (**d**) is spinach viral diseases; (**e**) is romaine lettuce downy mildew; (**f**) is romaine gray mold; (**g**) is romaine soft rot disease; (**h**) is romaine viral disease; (**i**) is chinese cabbage downy mildew; (**j**) is chinese cabbage black rot; (**k**) is chinese cabbage soft rot; (**l**) is chinese cabbage black spot disease; (**m**) is cabbage downy mildew; (**n**) is cabbage black spot disease; (**o**) is cabbage sclerotic disease; (**p**) is cabbage black rot disease; (**q**) is leaf lettuce downy mildew; (**r**) is leaf lettuce brown spot disease; (**s**) is leaf lettuce sclerotic disease; (**t**) is leaf lettuce powdery mildew.

During data collection, high-resolution digital cameras were used to ensure image clarity and detail preservation. The equipment included DSLR cameras from Nikon and Canon. Image acquisition was primarily conducted in the morning and afternoon to avoid any overexposure caused by strong midday sunlight. Each disease was systematically documented, covering the different stages of infection—from early onset to severe infection—to capture the progression of each disease comprehensively. Additionally, online images were collected to supplement and enhance the dataset. The selection criteria for the online images included high resolution, clear disease features, and reliable sources, as shown in Figure 1.

All images collected underwent rigorous screening and quality control to exclude unclear or mislabeled samples. Each image was accompanied by detailed metadata, including collection date, location, camera model, shooting parameters (such as aperture, shutter speed, ISO), and disease type. These details are crucial for subsequent data processing and model training, as they help researchers understand how background and shooting conditions may influence disease recognition. Regarding disease characteristics, detailed records were maintained for each type of disease manifestation on leaves. Downy mildew often appears as white powdery spots on leaves, significantly impairing photosynthesis and thriving under humid conditions. White rust causes white or pale yellow powdery spots on the underside of leaves, leading to leaf brittleness and wilting. Anthracnose forms dark brown to black lesions, often circular or irregular, which can cause leaf wilting when severe. Viral diseases result in stunted growth, deformation, or mosaic patterns on leaves, seriously affecting crop yield and quality. Gray mold manifests as gray, rotting patches on leaves, usually spreading from the edges inward. Soft rot causes water-soaked, mushy decay in plant tissues and thrives in warm and humid conditions. Black rot primarily affects leaf veins, causing blackening and necrosis that spreads along the veins. Black spot leads to circular or irregular black lesions on leaves, often surrounded by yellow halos. Sclerotinia survives in plant tissues as sclerotia, creating lesions and hardening infected parts. Brown spot appears as brown lesions, typically darker in the center and lighter at the edges, affecting the overall appearance of the plant. Powdery mildew forms a white powdery fungal layer on leaf surfaces, not only affecting the plant’s appearance but also weakening its growth.

### 4.2. Data Augmentation

In deep learning tasks, particularly when datasets are limited, data augmentation techniques play a crucial role in improving model performance. Data augmentation generates additional training samples by applying various transformations to the original data, thereby enhancing model generalization and reducing the risk of overfitting. Traditional augmentation methods, such as rotation, scaling, cropping, and flipping, have been widely adopted. However, with the advancement of deep learning, innovative augmentation methods like Cutout, Mixup, and CutMix have gained significant attention. These methods employ distinct transformation strategies to further diversify datasets, achieving remarkable performance improvements across various tasks.

#### 4.2.1. Cutout

Cutout is a simple yet effective image augmentation method. Its core idea involves randomly selecting a rectangular region in the input image and setting the pixel values within that region to zero. This technique simulates occlusion scenarios, compelling the network to learn more robust features instead of overly relying on specific regions of the image. A key advantage of Cutout is its computational efficiency, requiring only local masking during training. The operation of Cutout can be expressed as follows:

(9) $$I^{\prime }=I⊙\left(1-M\right)$$

where *I* denotes the original image, $I^{\prime }$ represents the augmented image, and *M* is a binary mask of the same size as the input image. The mask *M* generates a rectangular region at a random position, with a value of 1 indicating retained pixels and 0 indicating masked pixels. By introducing occluded regions, Cutout enhances the network’s ability to learn features across various areas, particularly under conditions involving partial occlusion or noise.

#### 4.2.2. Mixup

Mixup is a mixing-based augmentation method that creates new training samples by blending two images in a specific ratio. This is achieved through a linear interpolation of the image pixels and the weighted averaging of their corresponding labels. The primary advantage of Mixup is its ability to improve the model’s robustness to boundary ambiguities, mislabeled samples, and small datasets. The operation of Mixup can be described as follows:

(10) $$\tilde{I}=\lambda I_{1}+\left(1-\lambda \right)I_{2}$$

(11) $$\tilde{y}=\lambda y_{1}+\left(1-\lambda \right)y_{2}$$

where $I_{1}$ and $I_{2}$ are two input images, $y_{1}$ and $y_{2}$ are their corresponding labels, and λ is a value sampled from a uniform distribution U(0,1), representing the blending ratio of the two images. By interpolating both the data and the labels, Mixup enhances the model’s robustness to ambiguous sample boundaries and mitigates overfitting.

#### 4.2.3. CutMix

CutMix is another innovative image augmentation method that generates new images by cutting and pasting regions between two images. Specifically, a rectangular region is randomly selected from image *A*, cut out, and pasted onto a random position in image *B*. The labels are adjusted using a weighted average approach. By incorporating different local regions into the image, CutMix enhances the model’s robustness and improves its ability to learn local image features. The operation of CutMix can be formulated as follows:

(12) $$I^{\prime }=M⊙I_{A}+\left(1-M\right)⊙I_{B}$$

(13) $$y^{\prime }=\lambda y_{A}+\left(1-\lambda \right)y_{B}$$

where $I_{A}$ and $I_{B}$ are two input images, $y_{A}$ and $y_{B}$ are their respective labels, *M* is a binary mask representing the rectangular region cut from image *A*, and $y^{\prime }$ is the augmented label. The value λ, sampled from a uniform distribution U(0,1), determines the proportion of the cut region. CutMix enhances the model’s understanding of local image regions and improves its ability to recognize combinations of diverse object features.

### 4.3. Proposed Method

**Figure 2:**
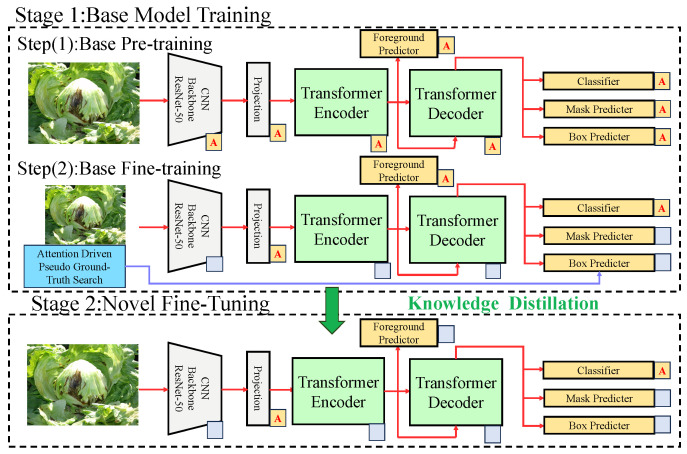
The proposed method consists of the following two stages: the base model training stage (Stage 1) and the new class fine-tuning stage (Stage 2). In Stage 1, multi-scale features are extracted from input images using a ResNet-50 backbone, and global contextual features are generated through a Transformer encoder and decoder. The object detection and segmentation modules are composed of a foreground predictor, a classifier, a mask generator, and a bounding box generator. In Stage 2, attention-guided pseudo-label generation and knowledge distillation strategies are utilized to further optimize the model’s adaptability and generalization for few-shot classes.The blocks labeled with ‘A’ indicate attention-enhanced components, where attention mechanisms are applied to improve feature selection and region activation. The gray blank blocks represent projection layers that map CNN-extracted features into the Transformer encoder’s latent space before further processing.

The proposed few-shot learning model for leafy vegetable disease detection and segmentation consists of multiple modules, as illustrated in Figure 2.

The overall process begins with the extraction of features from the input data, followed by task-specific processing, attention enhancement, and loss optimization. The preprocessed data are first passed through the backbone feature extraction module, which is built on the ResNet-50 CNN structure. Through multiple convolutional and pooling operations, the multi-scale features are extracted from the input images. The extracted features are then fed into the Transformer encoder, where global semantic representations are generated using the self-attention mechanism. The encoded features are subsequently decoded by the Transformer decoder, which connects to the dual-task modules for object detection and semantic segmentation. In the object detection branch, the decoder’s features are used to generate candidate bounding boxes and predict object categories. Accurate object locations are produced using regression formulas. In parallel, the segmentation branch employs a mask predictor to produce fine-grained segmentation masks for diseased regions, with mask generation relying on the activation of disease-relevant pixels via the attention mechanism. The attention-driven prototype module, denoted by the ‘A’ labels, refines feature representation by dynamically reweighting extracted feature maps, ensuring improved disease segmentation and classification. Additionally, the gray projection layers provide a transition between the convolutional feature maps and the attention-based Transformer pipeline.

To further enhance detection and segmentation performance, a prototype extraction module is utilized to extract prototype vectors for each disease class from the feature space. These prototype vectors are then incorporated into a prototype attention mechanism, which uses them to reweight the model’s feature maps, amplifying the feature responses for diseased regions. While the dataset contains 1000 to 2000 images per disease, our approach simulates few-shot learning by restricting the number of samples available to the model during training episodes. Specifically, the model is designed to generalize from only a handful of samples per class at a time, aligning with the meta-learning principles commonly employed in few-shot learning paradigms. Finally, the model is optimized using a multi-task loss function comprising detection loss, segmentation loss, and prototype loss. This approach allows the model to handle new disease variations with minimal labeled data, making it particularly useful for real-world agricultural applications where annotated disease images may be scarce or imbalanced. This comprehensive process ensures an efficient end-to-end pipeline, enabling the precise detection and segmentation of leafy vegetable diseases even under limited supervision conditions.

#### 4.3.1. Few-Shot Network Based on Detection and Segmentation

**Figure 3:**
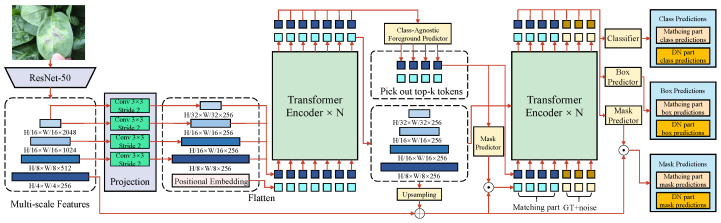
The network uses ResNet-50 as the backbone to extract multi-scale feature maps through multiple convolutional operations (3×3 kernel, stride of 2), such as H/32×W/32×256 and H/16×W/16×256. Positional embeddings are applied to the feature maps, which are then fed into the Transformer encoder for global feature modeling. The decoding stage includes a foreground predictor, a classifier, a bounding box generator, and a mask generator. The foreground predictor selects the top *k* most salient tokens, predicting class labels, bounding box locations, and pixel-level masks. Finally, the masks are restored to their original resolution through an upsampling module. This architecture supports the dual tasks of object detection and semantic segmentation and incorporates few-shot optimization strategies to enhance generalization for scarce classes.

An FSL-based network for object detection and segmentation is designed, as illustrated in Figure 3, to address the issue of data scarcity in leafy vegetable disease detection and segmentation. The architecture integrates multi-scale feature extraction, a Transformer-based encoder–decoder mechanism, and prototype learning to enhance performance under low-shot conditions.

The backbone of the network employs ResNet-50, which progressively reduces the input image resolution to H/32×W/32 through multiple convolution operations, such as 3×3 convolution kernels with a stride of 2. Positional embeddings are added to the extracted feature maps at each layer to preserve spatial information. Multi-scale feature extraction then generates feature maps of varying resolutions to accommodate the diversity and complexity of leafy vegetable diseases. The feature dimensions are defined as H/32×W/32×256, H/16×W/16×256, H/8×W/8×256, and H/4×W/4×256, providing rich contextual information for the subsequent detection and segmentation tasks. The Transformer encoder models these multi-scale features globally, processing the input features using a self-attention mechanism. The attention mechanism computes query (*Q*), key (*K*), and value (*V*) matrices, as in the following formula:

(14) $$\mathrm{Attention}\left(Q,K,V\right)=\mathrm{softmax}\left(\frac{QK^{T}}{\sqrt{d_{k}}}\right)V,$$

where $d_{k}$ denotes the scaling factor for feature dimensions to stabilize training. After *N* layers of the Transformer encoder, globally aware features are outputted and passed to the decoder for the detection and segmentation tasks. The decoder comprises a class-agnostic foreground predictor and a box predictor. The most salient *k* tokens are selected from the global features, representing the potential disease regions. These tokens are passed through a classifier to predict disease categories and a box predictor to regress bounding box coordinates. The classification and bounding box regression losses are defined as follows:

(15) $$L_{\mathrm{cls}}=-\sum_{i=1}^{N}\left(y_{i}\cdot \log\left({\hat{y}}_{i}\right)+\left(1-y_{i}\right)\cdot \log\left(1-{\hat{y}}_{i}\right)\right),$$

(16) $$L_{\mathrm{bbox}}=\sum_{i=1}^{N}{\left({\hat{x}}_{i}-x_{i}\right)}^{2}+{\left({\hat{y}}_{i}-y_{i}\right)}^{2},$$

where $y_{i}$ and ${\hat{y}}_{i}$ are the ground truth labels and predicted probabilities, respectively, while $x_{i},y_{i}$ and ${\hat{x}}_{i},{\hat{y}}_{i}$ are the ground truth and predicted coordinates of bounding boxes. To enhance the fine-grained segmentation performance, the mask predictor generates pixel-level segmentation masks from multi-scale features. The masks are upsampled to the original resolution using an upsampling module. The mask loss is defined as a binary cross-entropy loss, as follows:

(17) $$L_{\mathrm{mask}}=-\sum_{i=1}^{N}\left(m_{i}\cdot \log\left({\hat{m}}_{i}\right)+\left(1-m_{i}\right)\cdot \log\left(1-{\hat{m}}_{i}\right)\right),$$

where $m_{i}$ and ${\hat{m}}_{i}$ denote the ground truth and predicted pixel masks, respectively. Additionally, a prototype learning mechanism is incorporated, where the mean embeddings of each class are computed to generate class prototypes. These prototypes are used to construct an attention matrix, enhancing the model’s response to disease-relevant features. The prototype loss function is designed as follows:

(18) $$L_{\mathrm{prototype}}=\sum_{i=1}^{N}\left(||f\left(x_{i}\right)-C_{y_{i}}{\left|\right|}^{2}-\sum_{j\neq y_{i}}||f\left(x_{i}\right)-C_{j}{\left|\right|}^{2}\right),$$

where $f(x_{i})$ represents the embedding of sample $x_{i}$, $C_{y_{i}}$ is the prototype vector of the ground truth class $y_{i}$, and $C_{j}$ represents the prototype vectors of other classes. This mechanism ensures strong generalization and robustness under low-shot conditions. This design addresses several challenges in leafy vegetable disease detection. First, multi-scale feature extraction captures the morphological diversity of diseases. Second, the Transformer’s global modeling capability mitigates interference from complex backgrounds. Finally, the prototype learning mechanism improves feature representation with limited samples, enhancing detection and segmentation performance. Thus, this network provides an efficient and reliable solution for low-shot scenarios in agricultural disease control.

#### 4.3.2. Prototype Extraction Module and Prototype Attention

**Figure 4:**
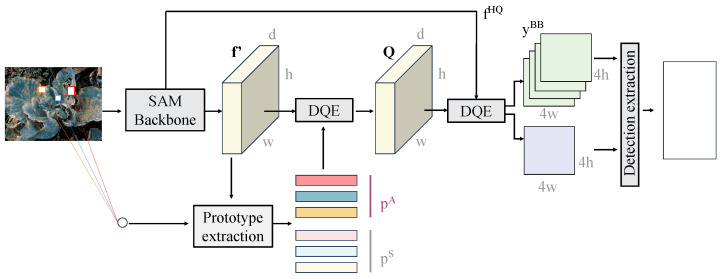
The module extracts class prototypes from the multi-scale feature maps output by the backbone through dimensionality reduction and clustering operations, generating prototype vectors for each class. The prototype vectors are correlated with the embeddings of the input feature maps through the prototype attention mechanism, resulting in an attention weight matrix that is used to reweight the feature maps. The reweighted feature maps are further fed into the object detection and segmentation modules, enhancing the feature response for diseased regions. This module effectively addresses class confusion and background interference in FSL by leveraging the global representation capability of class prototypes and the feature focusing ability of the attention mechanism.

The prototype extraction module and prototype attention mechanism, as illustrated in Figure 4, are designed to generate class-specific prototype representations through few-shot feature learning. These prototypes are used to guide the model’s focus on critical disease regions, thereby enhancing the accuracy and robustness of disease detection and segmentation.

The prototype extraction module retrieves high-dimensional embeddings from the feature maps extracted by the backbone network. The dimensions of the input features are H×W×C, where *H* and *W* represent the height and width of the feature map, and *C* represents the number of channels. For this study, the output feature maps from the backbone network include two levels—H/4×W/4×256 and H/8×W/8×512. These features undergo dimensionality reduction and clustering operations to generate class-specific prototype vectors.

The prototypes are derived by averaging the embeddings of samples belonging to the same class, ensuring that the prototypes capture the global feature distribution of each class. Once the prototype vectors are generated, they are passed to the prototype attention mechanism to reweight the feature maps. The core of the attention mechanism lies in calculating the correlation between the feature map F and the prototype vectors C to generate attention weights, as follows:

(19) $$A_{i,j}=\mathrm{softmax}\left(F_{i}\cdot C_{j}\right),$$

where $F_{i}$ represents the embedding at position *i* of the feature map, $C_{j}$ represents the prototype vector of class *j*, and $A_{i,j}$ is the attention weight between position *i* and class *j*. This mechanism assigns higher weights to regions of the feature map that are relevant to the target class, thereby enhancing the model’s focus on disease-specific regions. The attention weights are then used to reweight the original feature map, as expressed by the following equation:

(20) $$F^{\prime }=F⊙A,$$

where ⊙ denotes element-wise multiplication, and $F^{\prime }$ represents the attention-weighted feature map. The reweighted feature map is subsequently fed into the object detection and segmentation modules for further processing. This design offers several mathematical advantages. By computing the prototypes as the mean of sample embeddings, the inter-class distributional variance is significantly reduced, improving the representation capability for few-shot classes. The attention mechanism further aligns the feature map’s distribution with the target class, enabling the model to concentrate on relevant regions while suppressing background noise.

From a computational perspective, assuming the feature map contains *N* positions, each with a feature dimension of *D*, and the prototype vectors are represented as $C\in R^{K\times D}$, where *K* is the number of classes, the computational complexity of the prototype attention mechanism is O(N·K·D). This complexity is lower than that of the standard self-attention mechanisms, making it well suited for large-scale feature maps. In the context of leafy vegetable disease detection, this module effectively addresses several challenges. First, the prototype extraction module reduces reliance on large-scale annotated data by efficiently clustering features from limited samples. Second, the prototype attention mechanism strengthens the model’s response to disease-specific regions, particularly in complex backgrounds. Third, by combining global feature representations with attention mechanisms, the model demonstrates improved robustness in handling FSL scenarios and class imbalance. These attributes make the proposed approach an efficient solution for disease detection and segmentation in data-scarce agricultural environments.

#### 4.3.3. Prototype Loss Function

A prototype loss function is designed to address the challenges of data scarcity and class confusion in FSL scenarios. This loss function incorporates the prototype extraction module and prototype attention mechanism to enforce prototype matching constraints, enhancing the class distinctiveness and robustness in the feature space. Compared to the traditional cross-entropy and regression loss functions, the prototype loss function provides stronger discriminative power and adaptability. The core idea of the prototype loss function is to optimize the distance relationships between sample embeddings and their corresponding class prototypes, ensuring that samples are closer to their true class prototypes while being farther from the prototypes of other classes.

In the loss computation, the first term, $||f\left(x_{i}\right)-C_{y_{i}}{\left|\right|}^{2}$, minimizes the distance between the sample and its corresponding class prototype, while the second term, $\sum_{j\neq y_{i}}||f\left(x_{i}\right)-C_{j}{\left|\right|}^{2}$, maximizes the distance between the sample and other class prototypes. By optimizing this loss function, the model learns a feature space with higher inter-class separability and intra-class compactness. Compared to traditional cross-entropy and regression losses, the prototype loss function offers several notable advantages, including the following:

1. Structural constraints in feature space: Traditional cross-entropy loss optimizes classification probability distributions but does not directly optimize sample distributions in the feature space. The prototype loss function explicitly guides feature distribution through distance-based optimization.
2. Adaptability to FSL: Traditional loss functions rely heavily on large-scale labeled data, whereas the prototype loss function compresses sample feature information via prototype representation, making it more effective in few-shot scenarios.
3. Enhanced robustness: In cases of inter-class data confusion or label imbalance, the prototype loss function directly optimizes feature space distributions, mitigating the impact of class confusion.

The prototype loss function operates in conjunction with the prototype extraction module and prototype attention mechanism, forming a comprehensive optimization pipeline for FSL. The prototype vector $C_{i}$ is not only used to guide feature reweighting in the prototype attention mechanism but also to calculate the distance in the prototype loss function.

In this process, the optimization of the prototype loss function synergizes with the attention mechanism to ensure the separability of feature distributions and the relevance of class relationships. Assuming the feature space satisfies a manifold hypothesis, where each class’s data distribution forms a connected sub-manifold in the feature space, the prototype vector $C_{i}$ can be regarded as the center of the class manifold. By optimizing the prototype loss function, the embedding $f(x_{i})$ of each sample is gradually pulled towards the center of its corresponding manifold while being pushed away from the other manifolds. This optimization process improves intra-class compactness and inter-class separability in the feature space, significantly reducing classification confusion. In the context of leafy vegetable disease detection, the class feature distributions are often highly confused due to background interference and data scarcity. The prototype loss function effectively addresses these challenges. First, the optimization of prototype vectors establishes clear class boundaries in the feature space. Second, by integrating the prototype extraction module and prototype attention mechanism, the model demonstrates enhanced feature representation capabilities under few-shot conditions. Third, the loss function’s construction ensures robustness against class confusion, thereby improving the model’s detection and segmentation performance. These characteristics make the prototype loss function highly valuable, both theoretically and practically, in the task of agricultural disease detection.

### 4.4. Experimental Configuration

#### 4.4.1. Hardware and Software Platforms

The experiment was conducted on a high-performance computing platform equipped with a GPU-accelerated deep learning environment. Specifically, an NVIDIA RTX 3090 GPU (Santa Clara, CA, USA) was utilized to enable the efficient training and inference of deep neural networks. The system was built on PyTorch 2.4.1, leveraging its GPU acceleration capabilities through CUDA and cuDNN to enhance computational efficiency. For software and environment management, Anaconda was used to maintain consistency across dependencies, ensuring experiment reproducibility. Python 3.13.2 served as the primary programming language, with libraries such as NumPy, Pandas, and OpenCV employed for data processing and visualization. TensorBoard was used to monitor training progress and performance metrics. The entire setup was designed to ensure scalability and reproducibility, allowing experiments to be efficiently replicated in different environments.

#### 4.4.2. Hyperparameter Settings and Training Conditions

For the FSL task in agricultural disease detection, a series of suitable hyperparameter settings were adopted to ensure optimal performance on limited training data. The learning rate was identified as a critical hyperparameter, determining the step size for parameter updates during training. A high learning rate may cause oscillations around the optimal solution, while a low learning rate can result in slow convergence or getting trapped in local optima. In this study, the learning rate α was set to 0.001, which has demonstrated effective convergence in most neural network training scenarios. A learning rate decay strategy was implemented, reducing the learning rate by a factor of 0.1 every 10 epochs to improve stability and guide the model toward a global optimum.

The batch size was set to 16, meaning that 16 images were used for gradient updates in each training iteration. A smaller batch size reduces memory overhead and improves gradient estimation accuracy, particularly with smaller training datasets, helping to mitigate overfitting. During data preprocessing, all images were resized to 224×224 to meet the input requirements of CNNs. The dataset was divided into 80% training data, 10% validation data, and 10% test data. The training set was used to train the model, the validation set for hyperparameter tuning and model selection, and the test set for evaluating the model’s performance in practical applications.

To further enhance the model’s generalization capability and address potential instability caused by uneven data splits, 5-fold cross-validation was employed. The entire dataset was divided into 5 subsets, with 4 subsets used for training and the remaining subset for validation in each iteration, repeated across 5 cycles. The final model performance was calculated as the average of the 5 experimental results, reducing the influence of randomness and improving stability and reliability. To prevent overfitting, regularization techniques such as L2 regularization (weight decay) and Dropout were applied. The coefficient for L2 regularization was set to 0.0001, and the Dropout rate was set to 0.5, meaning 50% of the neurons were randomly dropped during training to improve model robustness.

The number of training epochs was set to 50. Cross-entropy loss was selected as the loss function, effectively measuring the difference between the predicted results and the ground truth in classification tasks. The Adam optimizer was used for optimization, as it provides adaptive learning rates and fast convergence. The hyperparameters for the Adam optimizer were configured as $\beta_{1}=0.9$, $\beta_{2}=0.999$, and $\epsilon =10^{-8}$, which have been proven effective across various tasks.

### 4.5. Baseline Models

Several state-of-the-art object detection and semantic segmentation models were selected as baselines for comparison, including YOLOv9 [56], YOLOv10 [57], DETR (Detection Transformer) [58], SegNet [46], TinySegformer [55], and MaskRCNN [59]. These models represent the diverse directions and advancements in object detection and semantic segmentation.

For object detection, the YOLO series (specifically YOLOv9 and YOLOv10) was chosen for its efficiency and real-time detection capabilities. YOLO uses a single CNN to simultaneously perform object classification and localization. YOLOv9 improves detection accuracy with deeper network architectures and optimized training strategies, while YOLOv10 further enhances inference speed and accuracy, particularly in small object detection. DETR employs a novel Transformer-based architecture, replacing traditional convolutional designs with self-attention mechanisms to capture relationships between positions in the image. By incorporating query mechanisms and precise bounding box localization, DETR improves recognition in complex scenes.

For semantic segmentation, SegNet, TinySegformer, and MaskRCNN were included as benchmarks. SegNet is a classic encoder–decoder-based network that compresses and reconstructs input images to perform pixel-level classification. Its lightweight design and low computational cost make it suitable for resource-constrained scenarios. TinySegformer, a lightweight Transformer-based model, leverages self-attention mechanisms to achieve pixel-level classification. Unlike the traditional CNNs, TinySegformer captures long-range dependencies, demonstrating superior performance in segmentation tasks with complex backgrounds. MaskRCNN, a widely-used method for instance segmentation, extends Faster R-CNN with an additional branch for generating segmentation masks for each detected object. MaskRCNN excels in precise object detection and pixel-level segmentation, making it ideal for complex instance segmentation tasks.

### 4.6. Evaluation Metrics

In this study, accuracy, precision, recall, and mAP were selected as the evaluation metrics. Accuracy is one of the most straightforward evaluation indicators, representing the proportion of correctly predicted samples among all samples. In classification tasks, accuracy is used to measure the proportion of correct predictions among the total number of predictions. Precision is another important metric that reflects the proportion of true positive samples among all samples predicted as positive by the model. Recall focuses on the model’s ability to capture positive samples, indicating the proportion of true positive samples correctly identified by the model out of all actual positive samples. Recall emphasizes “finding all positive samples”, aiming to predict as many positive samples as possible.

mAP combines precision and recall to comprehensively evaluate the model’s performance across different categories and detection thresholds. The calculation of mAP requires first plotting the precision–recall (PR) curve for the predictions of each category and then computing the area under the curve to obtain the average precision (AP) for each category. Finally, mAP is calculated as the mean of the AP values across all categories. The formulas for these metrics are as follows:

(21) $$\mathrm{accuracy}=\frac{TP+TN}{N}$$

(22) $$\mathrm{precision}=\frac{TP}{TP+FP}$$

(23) $$\mathrm{recall}=\frac{TP}{TP+FN}$$

(24) $${\mathrm{AP}}_{i}=\int_{0}^{1}\mathrm{precision}\left(r\right)\;dr$$

(25) $$\mathrm{mAP}=\frac{1}{N}\sum_{i=1}^{N}{\mathrm{AP}}_{i}$$

In these equations, TP represents the number of true positives, TN represents the number of true negatives, *N* denotes the total number of samples, FP represents the number of false positives, and FN represents the number of false negatives. precision(r) is the precision corresponding to recall *r*, and *N* denotes the number of categories.

## 5. Conclusions

The main objective of this study is to propose an efficient model for leafy vegetable disease detection and segmentation based on FSL and the prototype attention mechanism. With the increasing diversity of diseases and the complexity of backgrounds in agricultural production, the traditional disease detection methods face numerous challenges, including data scarcity, feature confusion, and background interference. Therefore, the ability to effectively identify different diseases with limited labeled data while improving model robustness and accuracy has become an urgent research problem. This study significantly enhances model performance in leafy vegetable disease detection and segmentation by introducing the prototype attention mechanism, combined with optimization strategies for FSL, especially excelling in complex backgrounds and few-shot conditions. The key innovations of this study are threefold. First, a FSL method combined with prototype attention mechanism is proposed, which generates global prototype vectors for each disease category through the prototype extraction module and uses attention mechanisms to guide the weighting of feature maps, significantly improving the model’s focus and feature response in complex backgrounds. Second, a prototype loss function is designed, which adjusts the distance relationships between the samples and the category prototypes during optimization, enhancing class separability and background interference suppression. Future research can further optimize this model and extend it to more agricultural scenarios to promote the development of smart agriculture technologies and improve the automation level of agricultural production.

## Figures and Tables

**Table 1:** Disease detection results on object detection task.

Model	Precision	Recall	Accuracy	mAP@50	mAP@75
DETR [50]	0.83	0.79	0.80	0.81	0.80
YOLOv9 [51]	0.85	0.82	0.83	0.84	0.83
YOLOv10 [52]	0.88	0.85	0.86	0.87	0.86
proposed method	0.93	0.90	0.91	0.91	0.90

**Table 2:** Disease detection results on segmentation task.

Model	Precision	Recall	Accuracy	mAP@50	mAP@75
SegNet [53]	0.87	0.82	0.85	0.84	0.85
MaskRCNN [54]	0.89	0.84	0.87	0.86	0.87
TinySegformer [55]	0.92	0.89	0.90	0.91	0.90
proposed method	0.95	0.92	0.93	0.92	0.92

**Table 3:** Disease detection results for various disease types.

Disease Type	Precision	Recall	Accuracy	mAP@50	mAP@75
Vegetable-Downy Mildew	0.96	0.93	0.95	0.94	0.93
Lettuce-Gray Mold	0.95	0.92	0.94	0.93	0.92
Chinese Cabbage-Black Rot	0.94	0.91	0.93	0.92	0.91
Cabbage-Sclerotinia	0.93	0.90	0.92	0.91	0.90
Romaine Lettuce-Brown Spot	0.92	0.90	0.91	0.91	0.90

**Table 4:** Ablation experiment on different attention mechanisms.

Disease Type	Precision	Recall	Accuracy	mAP@50	mAP@75
Standard Self-Attention	0.73	0.70	0.72	0.72	0.71
CBAM	0.85	0.81	0.83	0.84	0.83
Proposed Method	0.95	0.92	0.93	0.92	0.92

**Table 5:** Ablation study on different loss functions.

Disease Type	Precision	Recall	Accuracy	mAP@50	mAP@75
Cross-Entropy Loss	0.67	0.64	0.65	0.66	0.65
Focal Loss	0.84	0.80	0.82	0.81	0.81
Proposed Method	0.95	0.92	0.93	0.92	0.92

**Table 6:** Image data for different vegetable diseases.

Vegetable	Downy Mildew	Disease2	Disease3	Disease4
Spinach	1843	White Rust (1624)	Anthracnose (1341)	Viral Disease (1568)
Lettuce	1985	Gray Mold (1757)	Soft Rot (1492)	Viral Disease (1775)
Chinese Cabbage	1203	Black Rot (1506)	Soft Rot (1648)	Black Spot (1437)
Cabbage	1191	Black Spot (1522)	Sclerotinia (1675)	Black Rot (1980)
Romaine Lettuce	1780	Brown Spot (1125)	Sclerotinia (1367)	Powdery Mildew (1092)

## Data Availability

Data are contained within the article.
